# Increasing access to CAR-T therapy: a case study of an academic hospital’s alternative innovation model

**DOI:** 10.1186/s13023-026-04320-7

**Published:** 2026-04-10

**Authors:** Adrián Alonso Ruiz, Erika Shinabargar, Iulia Slovenski, Kaitlin Large, Marcela Vieira, Suerie Moon

**Affiliations:** Global Health Centre, Graduate Institute of International and Development Studies, Chem. Eugène-Rigot 2, Genève, 1202 Switzerland

**Keywords:** Advanced therapies, Pharmaceutical innovation, Access to medicines, CAR-T therapies, Alternative innovation models, Academic development, Hospital exemption, Advance therapy medicinal products, Orphan drugs, Acute lymphoblastic leukemia

## Abstract

**Background:**

Increasing costs of drugs for rare diseases have raised concerns about health systems’ sustainability and equitable access to these therapies. Newer and highly effective orphan drugs, such as Chimeric Antigen Receptor T-Cell (CAR-T) therapies, highlight the need for alternative innovation models that can offer greater affordability and accessibility. This case study examines Hospital Clínic Barcelona’s (HCB) alternative innovation model to develop ARI-0001 (varnimcabtagene autoleucel), a novel CAR-T therapy for certain forms of leukemia, at a price two-thirds lower than comparable therapies from the pharmaceutical industry.

**Methods:**

We conducted background research, and performed twenty-one semi-structured interviews with HCB staff, representatives of regional Health Departments, the Spanish Ministry of Health and regulatory agency, academics, civil society and patient groups. We used a framework drawing on concepts from complex adaptive systems, incorporating the resources used (i.e., funding, knowledge, and manufacturing capacity), the practices implemented (i.e., knowledge management, access practices and transparency), and the rules and norms that shaped HCB’s interactions with other actors in the system. We then identified the concept of institutional logics as well-suited to explain how HCB’s model overcame various barriers.

**Results:**

HCB’s model builds from three competing institutional logics – healthcare, academic and industrial – while also addressing each logic’s weaknesses. The healthcare logic shaped HCB’s affordable pricing of ARI-0001, but also posed organizational challenges. The academic logic drove HCB’s willingness to share knowledge and technology with peers, but posed barriers to fund late-stage clinical trials and regulatory processes. Lastly, HCB adopted an industrial logic by identifying in-house regulatory expertise and developing partnerships for production. The hospital exemption clause, a European regulatory pathway that allows hospitals to develop and produce treatments under certain conditions, allowed HCB to span the boundaries of the three competing logics.

**Conclusions:**

Our findings underscore the potential of academic hospitals to develop more affordable advanced therapies, the importance of conducive regulatory frameworks, and the challenges that academic hospitals face to expand this model. Through increased regulatory and financial support to academic hospitals, strengthened coordination, and public funding with requirements for access, this model could achieve innovation with affordability in a cutting-edge technological area.

**Supplementary Information:**

The online version contains supplementary material available at 10.1186/s13023-026-04320-7.

## Background

Despite the very positive results treating different types of blood cancers [1, 2], high prices of CAR-T therapies and their limited global availability have become a significant societal challenge, highlighting the need for alternative innovation models and policies that can offer greater affordability and accessibility to these cutting-edge technologies.

All approved CAR-T therapies are designated as Orphan Drugs in Europe or the US (with the exception of Breyanzi in the EU, with three orphan designations withdrawn by the company) [3]. Treatments for rare diseases, which historically lacked commercial attractiveness because of the small population sizes targeted [4], have become in many instances commercial blockbusters after the implementation of orphan drug legislation in different countries and regions, which provided both incentives and allowed for high prices for these drugs [5]. The innovation model for rare disease therapies is characterized by a relatively greater presence of academic research centers and smaller biotechnology companies in the discovery and earlier development stages, with large pharmaceutical companies being more present in the later-stage development and commercialization stages. The high level of engagement of patient groups across the lifecycle of many orphan drugs, which helps address R&D, regulatory and market access challenges, is another characteristic of this innovation model [4–8].

Many of these characteristics are present in the CAR-T space. Clinical development of CAR-T therapies is densely populated by hospitals and academic centers [9–16], mostly from China and the US [10, 13], and the current innovation model relies on out-licensing to pharmaceutical companies for late-stage development and commercialization [9, 12, 16–18]. In this model, high prices of CAR-T therapies are justified by small patient populations, high production, post-marketing, and logistics costs, and enabled by the monopolies granted by Orphan Drug Designations and other exclusivity incentives [2, 19]. CAR-T production is challenging [12, 16, 20–22], slowly shifting away from centralized models (where one factory prepares the viral vector and/or the genetically modified cells), to decentralized models (where most stages of the production process can be performed within a network of hospitals), opening up the possibility of lower logistical barriers, costs, and timelines [12, 18, 20, 21, 23].

There is an emerging body of literature discussing the innovation and commercialization models of CAR-T therapies through a decentralized network of academic hospitals, which could theoretically address challenges related to affordability and unmet health needs [11, 12, 17, 18, 23–27]. This literature captures several challenges to this alternative innovation model, many of which are related to those stages traditionally linked to larger pharmaceutical companies, such as financing new production infrastructure, high operating and maintenance costs associated with industrial quality standards [9, 11, 12, 17, 25, 28], or regulatory processes, which can be burdensome, expensive and new for academic hospitals [9, 11, 16, 25, 29].

The critical role of appropriately flexible regulatory regimes has received relatively little attention with the exception of the literature focused on the hospital exemption (HE) clause [30, 31], a European regulatory pathway that facilitates the academic development of advanced therapy medicinal products (ATMPs) [9, 32], and improves the availability of drugs for indications that are not commercially viable [24, 25, 28, 29].

This clause exempts from centralized regulatory approval those ATMPs prepared on a “non-routine basis,” that are used in a hospital within the same Member State (MS), following the medical prescription for an individual patient, under certain quality standards. However, different interpretations of this clause across EU member states create diverging quality, safety, and efficacy requirements, different definitions of “non-routine basis”, or different limits on using the HE when therapeutic alternatives approved through the centralized procedure exist [11, 24, 25, 28, 33–36].

Although many of the challenges in developing academic CAR-T therapies are covered in the literature, there is not to our knowledge, an in-depth study of the development of an academic CAR-T therapy that sheds light on how developers address these challenges and how different actors (e.g., regulators, research funders) and sets of norms and rules influence the development of these alternative innovation models. This study delves into these factors by studying the development of ARI-0001 (varnimcabtagene autoleucel) by Hospital Clínic Barcelona (HCB) as an alternative innovation model that aims to couple innovation in a cutting-edge technology such as CAR-T, with greater affordability and access.

In February 2021, the Spanish Agency of Medicines and Medical Devices (AEMPS) approved the use of ARI-0001 under the HE clause, to treat adult patients with relapsed or refractory acute lymphoblastic leukemia, (R/R ALL), after a pilot study (NCT03144583) reported positive results, with Overall Survival (OS) of 68.6% and progression-free survival (PFS) at one year of 47% [37]. ARI-0001 was incorporated into the Spanish health system with a price under one-third of the cost of similar CAR-T therapies developed by the pharmaceutical industry [36, 38]. At the time of writing, ARI-0001 was undergoing a multi-centric phase two trial (NCT04778579) in Spain, and a comparative trial with axicabtagene ciloleucel (Yescarta) (NCT05641428) in the Netherlands. Furthermore, a consortium of European hospitals received funding to do a multi-country phase 2 clinical trial with ARI-0001 to submit the regulatory dossier for centralized approval at the European Medicines Agency (EMA) [39, 40]. Finally, in January 2025, varnimcabtagene autoleucel received regulatory approval in India after a phase 2 trial (CTRI/2022/03/041162) [41, 42], becoming the second CAR-T therapy approved in the country [43].

The remainder of this article explains how HCB developed ARI-0001, by identifying the resources HCB mobilized, the practices it adopted, and the norms and rules that shaped its interactions with other actors. We identify barriers and analyze how HCB overcame them by adopting strengths from three competing institutional logics – healthcare system, academic and industrial. We highlight strengths and weaknesses of this alternative innovation model, and conclude with recommendations for how the benefits of this model could be expanded and consolidated.

## Methods

This case study is one part of a broader research project, New Business Models for Governing Innovation and Access to Medicines (NBM), and adopts the project’s conceptual framework [44], which defines and conceptualizes the pharmaceutical innovation system as a Complex Adaptive System (Complex Adaptive Pharmaceutical Innovation System, CAPIS). We defined CAPIS as a group of actors that interact in a dynamic, sustained and non-linear way through time to develop a new pharmaceutical product. Norms and rules (e.g., intellectual property, social norms and expectations, regulatory frameworks) shape how these actors interact, the resources they use and have at their disposal, and the practices they engage in to render new outcomes (e.g., new affordable therapies) [44]. CAPIS are comprised of three types of actors: implementers or those doing R&D, funders or those funding R&D, and governing actors, or those influencing R&D by making, applying or interpreting relevant laws (e.g., regulators, legislators, patent offices), or seeking to advance or otherwise improve the R&D system (e.g., patient organizations, civil society groups).

We conducted background research on ARI-0001, then identified and contacted 23 relevant organizations for interviews (Table 1). The selection of interviewees was initially done through online searches to gather information about the history of ARI-0001 and was then complemented with snowball sampling. Of the 23 contacted organizations, we conducted 21 semi-structured interviews with 24 individuals from 13 organizations, while 10 organizations did not respond, refused to participate, or responded too late to be interviewed. The first interview took place in 2021 as part of the broader NBM research project. Six interviews were done in person, including four interviews during a field visit to HCB, and the other 15 were done online during 2023. Fifteen of the 21 interviews were conducted in Spanish and transcribed with Microsoft Word 365. The remaining six interviews were conducted in English and were transcribed using Otter. Only the excerpts used in this publication were translated into English. Interviews were performed until thematic saturation was reached.

**Table 1:** Summary table of actors identified

Group	Actor	Number of interviews	Number of interviewees
Implementer	Hospital Clínic Barcelona (development team, leadership, fundraising team)	6	6
	Other academic hospitals in Spain	1	1
	Spanish Network of Advanced Therapies (TERAV)	1	1
	Stichting Hemato-Oncologie voor Volwassenen Nederland (HOVON) (did not participate)	-	-
	Immuneel Therapeutics	4	6
	Pharmaceutical Industry (did not participate): Novartis, Bristol Myers Squibb	-	-
Funders	Fundació Glòria Soler	1	1
	Fundació La Caixa	1	1
	Instituto Carlos III (did not participate)	-	-
	Centre for the Development of Industrial Technology (CDTI) (did not participate)	-	-
Governing actors	Ministry of Health (service portfolio and reimbursement department)	1	1
	Spanish Agency of Medicines and Medical Devices (AEMPS)	1	1
	Catalan Department of Health	1	1
	Salud por Derecho	1	2
	Melanoma Patient Network Europe	1	1
	Academic #1: Expert in regulatory aspects of ATMPs Academic #2: academic ATMP developer	2	2

The analysis of the interviews was done following the framework method, a type of thematic analysis frequently used in qualitative research for health sciences [45, 46], where an analytical framework is created *ex ante* to code the interviews, and gets modified inductively as interviews are coded. The coding of the interviews was done by two researchers (AR for Spanish interviews and ensuring intercoder reliability and ES for interviews in English) using a framework based on the one used in the broader research project. One researcher (AR) reviewed the coding and ensured that the approach followed by the two coders was aligned through consensus meetings. Field notes and interview guides were kept in hand-written format and were used to structure the analysis.

Based on the analysis of the interviews, we inductively identified three different institutional logics or norms relevant to HCB’s alternative innovation model: healthcare, academic and industrial logics in the pharmaceutical sector. We have used the terms norms and logics interchangeably, given the similarities found in Lander’s definition of logics as the “implicit and socially shared rules of the game that describe behavior in a rule-like way, while being so entrenched in a social group that they become taken-for-granted as legitimate” [47, 48] and Hein and Moon’s definition of informal norms as those “based on shared norms and beliefs and not on formal institutional decisions”, that express “moral codices” and “help to create stable expectations with regard to behavior within the respective group, simplifying human interactions” [48]. We relied on the scholarship that has studied the relationship between healthcare and academic logics [47, 49–51], as well as the specific social norms that apply in the context of the Spanish healthcare system, which has a deep connection to notions of health equity, universality and accessibility [52]. We used Merton’s conceptualization of academic logics, based on principles of openness, disinterestedness and universalism [53] and the scholarship that explores the tensions between academic and commercial-industrial logics [54]. Finally, to define industrial logics in the pharmaceutical sector, we relied on the literature describing the norms and principles that guide the sector (e.g., its market-driven, profit-maximizing nature, its lack of transparency and equity), as well as the capacity of larger pharmaceutical companies to mobilize resources to drive the later stages of development and commercialization (e.g., the knowledge and capacity to scale-up production and clinical development, or the infrastructure for large-scale marketing, and distribution) [5, 6, 55, 56].

## Results

**Fig. 1:**
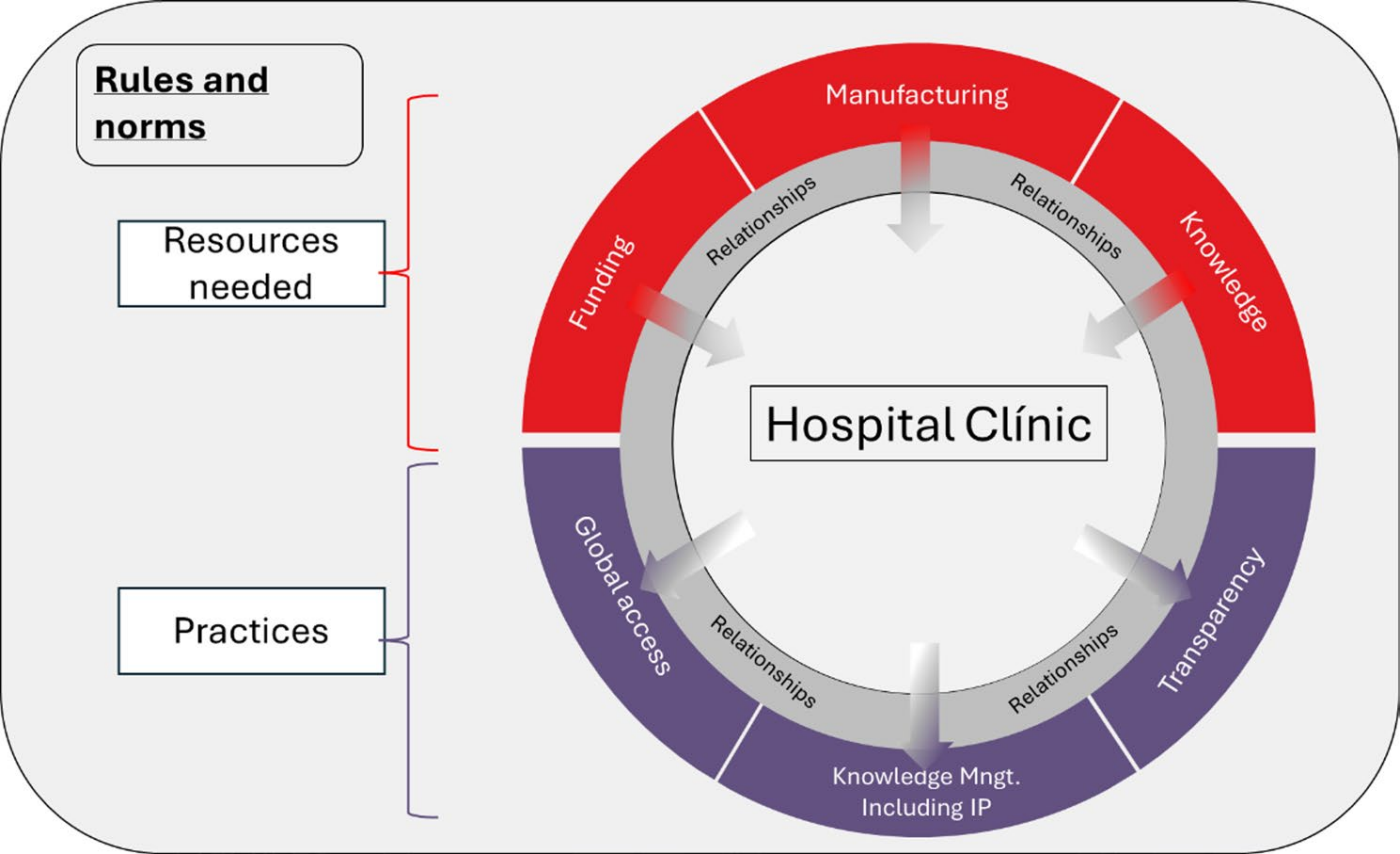
Analytical framework

**Fig. 2:**
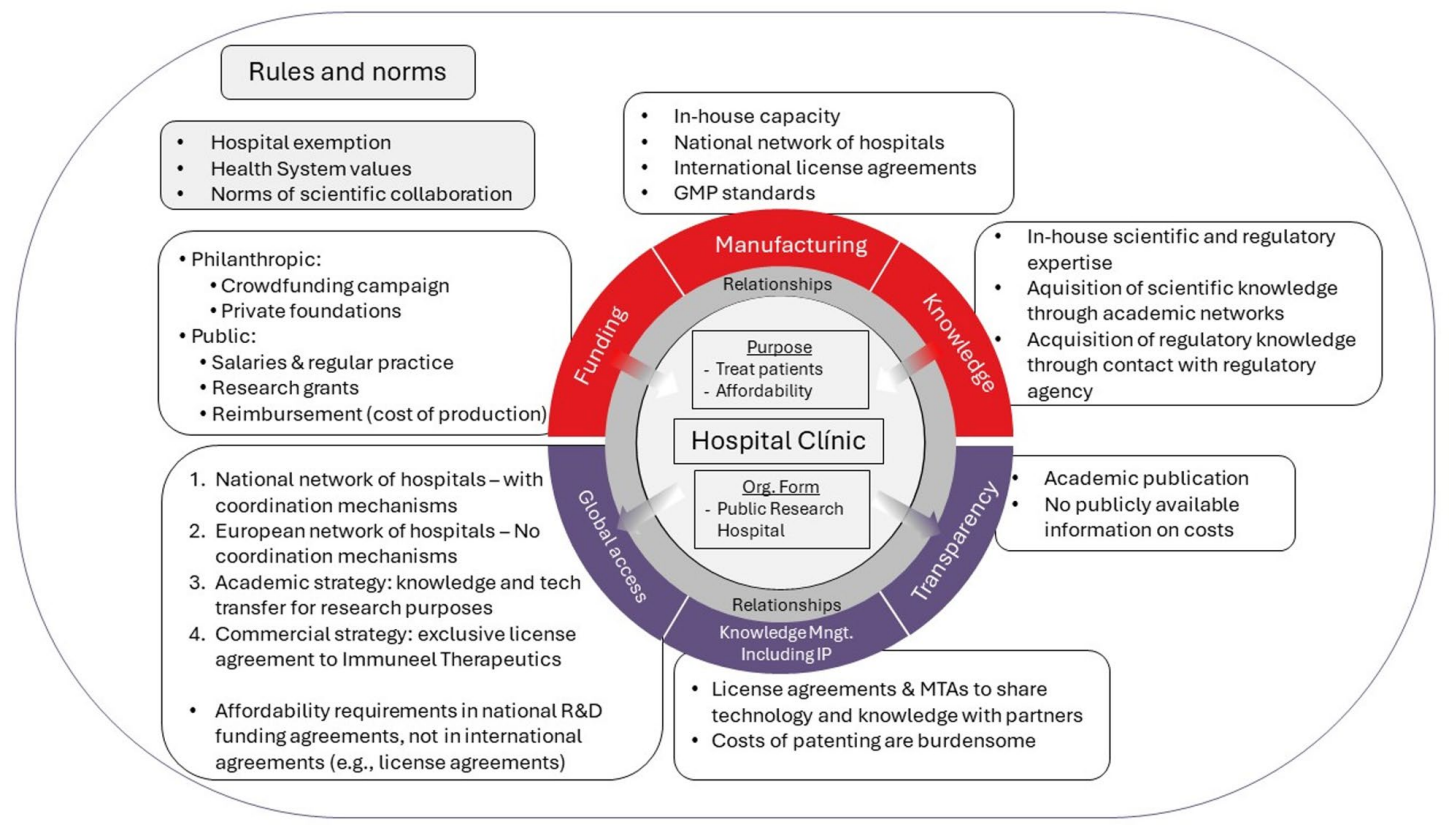
Descriptive diagram of HCB’s innovation model

We structured the results according to the analytical framework in Fig. 1. Firstly, describing the key characteristics of the central actor (i.e., HCB). Secondly, we analyzed how HCB mobilized the resources necessary to develop the product (i.e., knowledge, funding and manufacturing capacity), and the practices it adopted (i.e., access plans, transparency and knowledge sharing, including IP). Third, we identified the relevant rules (e.g., HE clause) and norms or logics (e.g., data sharing in academia, universality and access to healthcare in Spain) that shaped the development of ARI-0001. A summary of this analysis can be found in Fig. 2.

### Characteristics of the organization: mission, objectives and priority setting

HCB is a public academic hospital with an associated research center, the Fundació de Recerca Clínic Barcelona-Institut d’Investigacions Biomèdiques August Pi i Sunyer (FRCB-IDIBAPS). All HCB interviewees highlighted that their main motivation to develop ARI-0001 was to treat their patients with CAR-T therapies, in light of the results of clinical trials in the US and China during the 2010s [2]. As these trials had not taken place in Europe, it was impossible to access these treatments during their clinical development [24]. This motivation was later complemented with the objective of making the treatment more affordable to the healthcare system, with some interviewees highlighting that profit was never among their objectives (CS_1, CS_3, CS_10).

*Our intention was to be able to give patients more effective CAR-Ts than those they could not access. Then it has also evolved in a motivation to try to get CAR-Ts at a lower price than the commercial ones. - CS_1*

*I’m telling you! We are not looking to make money! - CS_3*

Transforming a healthcare institution to also be a pharmaceutical developer presented challenges that can be summarized as lack of a “business structure,” which creates problems setting research priorities, a clear direction or long-term view (CS_3, CS_12, CS_1).

Different researchers at the hospital had different research priorities and portfolios, which could translate into difficulties prioritizing research projects. In addition, some interviewees (CS_3, CS_1) sometimes questioned whether their decisions were correct (e.g., their decision to try to obtain regulatory approval themselves, or their approach to pricing), or if they were doing something that did not make sense, highlighting that although the team was strongly driven by their values and mission, the lack of clear strategic support to advance product development can be a limitation.

*We don’t have a professional background to know what’s right*,* what’s not right. Whether we do the right thing*,* or we go overboard or we do something dumb*,* […]. For that we need a professionalized infrastructure and […] a big hospital doesn’t have it. Because a hospital has a legal department or an accounting department*,* but if you ask them about the EMA*,* they don’t even know what you’re talking about. - CS_1*

Despite these challenges, collaborative work and engagement from the hospital leadership was essential to establish direction and strategy (CS_1, CS_2, CS_3, CS_10, CS_12).

*Within the hospital it has not been easy either. It is obviously the first time that we produce a medicine ourselves. The production piece needs to interact with the immunology piece*,* […]*,* with the clinical piece and the regulatory. Without the regulatory piece*,* all of this would have been impossible. So*,* coordinating these elements*,* the research-industrial*,* the clinic and the regulatory has been obviously difficult. But I think we have managed it. - CS_10*

### Resources

In alignment with the project’s conceptual framework, we describe below how HCB accessed the three kinds of resources necessary to develop ARI-0001: financing, manufacturing and knowledge.

#### Financing ARI-0001’s development

Financing the development of ARI-0001 was challenging, as there were no pre-existing funds allocated for this purpose. Although some of the doctors had received research grants in 2013, this funding was not enough to finalize the preclinical phase and do the first clinical trial. When she heard about the lack of funding in 2016, Ariana Benedé, a patient suffering from ALL, and her mother began “Project ARI”, a crowdsourcing campaign that was able to raise 1.8 million EUR between 2016 and 2018 from 23 associations and foundations, nearly 1500 individuals, and 56 private companies [25]. This supported the purchase of two automated production systems (CliniMACS Prodigy), funded two research fellowships in the US to learn the production and treatment procedures; helped finish the preclinical phase, and treated the first 15 patients in a phase 1 trial (CS_1, CS_8, CS_9).

In addition, philanthropic foundations contributed to the development of this therapy. For instance, Fundació La Caixa, a science and innovation funder in Spain and Portugal, contributed 5 million EUR over three years (2019–2022) in a funding agreement renewed in July 2023 for the 2023–2026 period [57].

The two philanthropic funders interviewed highlighted the relevance and innovative approach of this project as the main reason to fund it, despite not being part of their usual funding areas. Interviewees highlighted the flexibility and speed of philanthropic and crowdfunding initiatives that allowed HCB to purchase material, work and generate results in a faster and more efficient manner than through competitive public R&D funding schemes (CS_8).

However, public funders have also been essential in the development of ARI-0001. The Institute Carlos III (national health research funder) provided 2 million EUR for clinical research, in addition to a previous 300,000 EUR grant for basic research. The government of Catalonia provided a specific funding scheme consisting of 1.4 million EUR per year, during at least six years, dedicated to cover the costs of administering ARI-0001 to R/R ALL patients, prepare the CAR-Ts, hospitalization costs and supportive treatment (CS_1, CS_13).

As competitive research grants usually funded early-stage and preclinical stages, academic developers struggled to fund clinical trials, regulatory costs, or the coordination of the network of academic centers involved in CAR-T development (CS_3, CS_20).

One reason why these costs were not seen as a priority by public funders is because they have been traditionally covered by pharmaceutical companies, limiting researchers’ capacity to meet certain regulatory requirements (e.g., number of patients in a clinical trial), with the economic resources available.

*European grants are not designed for pivotal trials either*,* because academic centers have never done pivotal trials*,* because registration trials have always been done by the pharmaceutical industry. […] I can easily show [public funders] that it’s not a whim of mine*,* I can say*,* “No*,* look at this document that the EMA just sent us. It says that the 30 patients for which we have funding are not enough and that we need 70. And at least 1/4 of them have to come from centers outside of Spain. I will write the protocol for you. But who pays for it? In the end it is an economic barrier. - CS_3*

The development of ATMPs became a political priority for the Spanish government at that time, launching the “Strategic Project for Recovery and Economic Transformation on Vanguard Health” (PERTE Salud de Vanguardia in Spanish) funded through the European COVID-19 Recovery Plans. This project expanded research funding calls to cover clinical research, leveraging the national network of ATMP academic researchers and infrastructure, and aimed to attract private investors and companies to fund these late-stage expenses [58].

However, the entrance of private investment was not seen as desirable by some interviewees. Likely shaped by the negative views on marketization in the public healthcare system, some correlate accepting private investment with profiteering (CS_1, CS_3, CS_2, CS_8).

*[Accepting private investment] is to make a medicine more expensive so that an investor makes money out of a medical procedure. No way - CS_1*

At the time of writing, HCB overcame some of these financial barriers by turning to philanthropic funding, receiving in January 2024 a 9.3 million EUR grant from a consortium of five anti-cancer charities to do a multi-country phase 2 clinical trial in academic centers of five EU countries. This trial would allow HCB to submit the regulatory dossier for centralized approval at the EMA [39, 40].

#### Knowledge

In addition to financing, accessing relevant knowledge was essential for advancing ARI-0001. Two types of knowledge were fundamental to the interviewees: scientific and regulatory. The adjacent research center (IDIBAPS) allowed HCB to have solid scientific foundations and multidisciplinary teams in-house that enabled the development of CAR-T therapies.

Having regulatory expertise was identified as a success factor in the literature [10, 16] and among the interviewees (CS_3, CS_1, CS_10, CS_12, CS_6, CS_7). At HCB, two researchers had regulatory expertise at national and European levels, which facilitated the preparation of regulatory dossiers, clinical trial design, and reporting to the regulatory agency in “the same language”. Adopting a “regulatory mindset” helped to establish decision-making processes that considered the long-term strategy of obtaining regulatory approval, and the short-term view of treating individual patients.

*[The pharmaceutical regulatory system] is like a cult. If you agree to be in the cult*,* you are alright. But if you are not from the cult…someone needs to teach you how the cult works! - CS_2*

*You have to show [regulators] that you understand them*,* you need to be convincing and speak their language. And you need to understand how they think. And that is why it has been so important to have people that have worked there. - CS_3*

Regulators were a key external source of knowledge that HCB accessed. Contact with regulators provided HCB with technical and scientific support (e.g., which indications pursue, understand the regulatory requirements, discuss the adequacy of the data provided) (CS_3, CS_12, CS_20) [16], reducing inefficiencies and providing strategic guidance to continue the development, while providing HCB with regulatory knowledge.

*Regulatory agencies are very willing to help these initiatives. They are aware of the huge economic barrier that commercial CAR-T therapies pose*,* and they are seeing more clearly that the future might come this way. They are not lowering their standards*,* and they are really strict with us. As strict as they would be with [the industry] but while being strict*,* they help. I mean*,* before you… [do something*,* they tell you] ‘Do not do it like this*,* do it this other way’. - CS_3*

However, discussion on regulatory standards with regulators sometimes created tensions arising from the perception that regulatory requirements are tailored to pharmaceutical companies with large budgets (CS_2, CS_4, CS_12) [16].

*One of the aspects where we have some discrepancies with researchers has to do with the GMP inspections. [Academics]*,* sometimes*,* and not necessarily with ARI*,* find them very demanding and they think it is excessive for a small production*,* and that if you make a risk analysis*,* it would not be necessary to have such an exhaustive level of inspection and compliance. But of course*,* what we are demanding is what will make us be sure that the treatment is of quality. - CS_7*

In addition to accessing regulatory expertise, HCB had access to international academic networks, which influenced the successful development of ARI-0001. Two of the doctors in the team received fellowships to study the development and treatment standards in academic hospitals in the US where these therapies were being developed, bringing back that expertise and combining it with the in-house knowledge available. Interviewees highlighted the importance of sharing and accessing this knowledge to avoid duplication of efforts *(“it is possible that some of the things that we learnt there*,* allowed us to skip three or four tests” - CS_2*), innovate from pre-existing knowledge *(“one of the big changes that we introduced is that we were the first ones using a new bioreactor*,* no one had used it before to treat a patient” - CS_2)*, and learn standards of care *(“I needed to know if patients needed to go to the ICU*,* administering the treatments…” CS_3).*

This open way of sharing knowledge was linked to the norms of medical and academic environments – that is, an academic logic – as opposed to traditional pharmaceutical development environments where market competition means that knowledge is not usually transferred freely or openly.

*It has been like that for a long time in medicine. And in science too. But since all the patent and [intellectual] property protection stuff came in*,* it has become more aggressive. Because everything is linked to profit and business. […] We thought*,* ‘[it is going to be] similar to when a surgeon visits to learn a procedure. And in the first contact [the US hospital] told us that it would be like that. But afterwards the university sold [the treatment] and the rules changed. - CS_2*

*It’s quite normal. For me*,* in the non-profit scientific world*,* this is normal. In the industry world*,* it is not. - CS_3*

#### Manufacturing

HCB produced ARI-0001 in-house on a non-routine and individualized basis, following the hospital exemption clause. Interviewees from HCB expressed confidence in the hospital’s capacity to produce with high quality standards, acknowledging the need to acquire training and invest in certain infrastructures (CS_2, CS_3, CS_10, CS_12). The use of closed systems for production of CAR-T cells facilitated the standardization of production processes and was an innovative feature of the development of ARI-0001 [59]. Since the start of the development, HCB has increased its infrastructure, expanding the number of these bioreactors, and the number of people working in production [18]. Furthermore, with the expansion of the network of Spanish hospitals, more hospitals are able to produce the treatment, scaling-out the production of ARI-0001.

### Organizational practices

The previous sections addressed the resources that HCB had at its disposal to advance the development of ARI-0001; below we describe the practices HCB adopted to make the treatment more accessible to patients.

#### Knowledge- and technology-sharing practices

HCB planned to expand access to its products through four different pathways. All four relied on HCB’s willingness to transfer knowledge and technology to recipient organizations, while enforcing certain rights and conditions, such as exclusive use under clinical trial settings in certain cases, or the establishment of pharmacovigilance plans and reporting mechanisms. In exchange, the Hospital obtains data for the regulatory dossier, strengthens its partnerships and its scientific reputation.

However, there were differences depending on the type of actor and the existence of coordination mechanisms. Through a national network of hospitals, HCB was able to do a multi-centric clinical study by building partnerships with other Spanish hospitals, sharing the knowledge to modify the cells with HCB’s viral vector and administer them to patients. As these hospitals were mostly part of the healthcare system, prices in this model were expected to be more affordable to Spain’s healthcare system (CS_1, CS_6, CS_7, CS_10, CS_11, CS_20). This network existed formally (Spanish Network of Advanced Therapies, Red TERAV) which facilitated coordination and interaction among researchers.

HCB envisioned a similar network at the EU level, to facilitate sharing of scientific, regulatory or market-access knowledge [9, 11, 17, 28]. However, this presented many challenges, as there were no specific funding or governance mechanisms at European or international levels to facilitate the creation of such network (CS_1, CS_20). Nevertheless, there were some signs showing HCB’s success, such as the signature of a license agreement with HOVON, a Dutch foundation that carried out clinical trials in haemato-oncology in the Netherlands and Belgium [9, 11, 17, 28] (CS_1), or the 9.3 million EUR grant described in section “Financing ARI-0001’s development” to do a confirmatory trial with academic centers from 5 EU countries.

In addition to the creation of hospital networks, HCB aimed to share knowledge and technology with hospitals and academic centers outside of Europe that were still not able to carry-out clinical research. In different interviews, there were mentions of agreements with hospitals from Brazil, Colombia, Lebanon and Egypt, but there was no further publicly available information.

In 2020, HCB signed an exclusive license agreement with an Indian company, Immuneel Therapeutics, transferring the technology and knowledge to produce ARI-0001 and granting exclusive rights to commercialize in India [60]. Through this commercial model, Immuneel Therapeutics was responsible for clinical development, manufacturing, regulatory approval, and commercialization of the CAR-T in the country (which was approved in January 2025), and both parties were working together to expand commercialization rights in other countries in the region.

Immuneel Therapeutics’ mission was to provide CAR-T therapies at affordable prices in a country where internationally approved gene therapies were not available when the partnership began, and where the high demand and price sensitivity for this therapy could help bring prices down. It was unclear how affordability would be ensured, considering that the license text is unavailable, the expensive production processes, and the need to recover the investment made by the company (CS_18). Immuneel Therapeutics’ price for Qartemi (the name of ARI-0001 in India) ranges between Rs 35 lakh to Rs 50 lakh (EUR 33.947 and EUR 48.496, exchange rate as of 14th November 2025) per treatment [43]. However, interviewees did not provide further information on how the price is set. As with the above-mentioned collaborations, this agreement allowed HCB to use the data generated by Immuneel Therapeutics in the European regulatory dossier.

Coordinating and managing these agreements and networks was very resource intensive. Ensuring appropriate technology and knowledge transfer, the appropriate production and use of the treatment, and analyzing the data obtained in the clinical trials and pharmacovigilance reports, required resources that the hospital struggled to secure (CS_2, CS_12).

*This is an academic center*,* where research is a priority over a strategy of ‘I’m going to try to make the product data as reliable as possible [for regulators].’ And so*,* there are dozens*,* if not hundreds*,* of opportunities for academic collaborations: one hospital in Brazil*,* two in Chile…[…] [T]hey have no idea that you are the owner of the product and you are responsible for what is done with that product*,* […] so you have to make a contract. A very strict agreement*,* where they commit to use the product according to the standards that you set. […] they must commit to sending you all the safety data within the deadlines set by the regulators*,* etc. - CS_12*

These strategies often resulted from professional and personal connections, highlighting the importance of relationships for making this innovation model work. The license with Immuneel Therapeutics stemmed from the connection that a mutual acquaintance established between one of the doctors in HCB and the leadership of the company (CS_14). In this sense, the Hospital seemed to be flexible regarding its strategies to expand access.

Although intellectual property (IP) was not frequently mentioned during the interviews, HCB was undergoing a freedom-to-operate analysis to ensure that none of their practices or processes infringe on IP held by others. Some interviewees complained about the administrative costs of managing its patents, and how that interfered with their capacity to treat patients (CS_2).

#### Pricing

ARI-0001 was priced in Spain at EUR 89.270 per treatment [61], which is less than one-third of what similar products developed by pharmaceutical companies cost [3]. Researchers at HCB stated that this price reflects the cost of producing the treatment in Spain, and the Ministry of Health has published the standardized criteria they use to price non-industrial ATMPs, which include the costs of preparation, production, cleaning, and quality control, and a small premium calculated as a percentage of the cost of production [62], therefore excluding costs related to hospitalization or supportive treatments (Table 2).

**Table 2:** Cost breakdown used to price non-industrial ATMPs. Translated from the Spanish Ministry of health [62]

Category	Activity	Description of billable items
Preparation	Diagnostic Tests	• Cost of diagnostic tests required for the patient to obtain the Starting Material (SM).
	Drug Administration	• Costs of drugs used to stimulate hematopoietic progenitors.
Production	Obtaining SM	• Personnel time for collection, • Consumable materials used to obtain and preserve SM • Time employed in labeling/packaging and preparation of the SM.
	Reception of SM	• Personnel time for documentation review, traceability, and conditioning. • Cost of quality controls on the SM.
	Processing of SM	Sample preparation, isolation, selection, incubation, and cell proliferation (calculating average cost per batch/patient if multiple passages are required). Includes costs on: • Personnel time, • Packaging materials, • Fungible consumables, • Reagents, culture media and viral vectors • In-process controls.
	Cryopreservation	Applicable to SM, intermediates, or finished product. Costs include: • freezing/thawing time (including traceability), • Consumables and cryopreservation media • Maintenance cost per unit of time.
	Final Packaging	Personnel time and consumable materials for the final conditioning of the product.
	Transport	Time for packaging in validated transport equipment and documentation; transport costs if production occurs at a different center.
	Environmental Control	Personnel time for microbiological/environmental monitoring, consumables, and culture media employed.
Quality Control (QC)	Microbiological QC	Tests for sterility, mycoplasma, and endotoxins during various phases. Includes: • Personnel time, • Consumables and reagents.
	Final Product QC	Tests such as flow cytometry, viability, and cell count. Includes: • Personnel time, • Consumables, and reagents.
Batch Release	Technical Review	Time spent by the Technical Director reviewing product documentation for release.
Production Unit Costs	Qualification	Costs for qualification of the Production Unit, laminar flow cabinets, incubators, etc.
	Calibration	• Calibration of probes, measuring equipment, • Calibration of electromedical devices, • Calibration of particle counters, • Calibration of microbiology ovens.
	Maintenance	Maintenance of HVAC, air filtration, cabinets, incubators, fridges, freezers, gas detectors, etc.
	Aseptic Simulation	Cost of “Media-Fill” tests (performed twice yearly): personnel time, consumables, and culture media.
Cleaning & Sterilization	Cleaning of the Unit	• Cost of cleaning products per intervention • Personnel time for cleaning surfaces and the unit.
	Sterilization	• Time for material preparation/sterilization, • Media preparation, • Costs for cleaning/sterilizing personnel clothing.
Other Production Costs	Failures & Waste	• Costs inferred from production failures (% of total) • Costs of produced medications not used (due to disease progression, patient death, etc.).
	Depreciation	Depreciation of equipment acquisition, considering obsolescence and useful life.
Other costs (not included under production costs)	R&D premium	A progressive margin added to the total direct costs. • Tier 1: 5% (up to €20,000). • Tier 2: 3% (€20,001–€30,000). • Tier 3: 2% (€30,001–€40,000). • Tier 4: 1% (>€40,000).

The connection with the public healthcare system was essential to understand HCB’s perspective on pricing and access. For example, HCB requested the inclusion of language in the funding contracts with philanthropic funders, which stated that ARI-0001 would be delivered at affordable prices in the public healthcare system (CS_9, CS_11). Interviewees from the regulatory agency, the Ministry of Health, and the Spanish Network of Advanced Therapies also acknowledged that the investments made from the public sector should have a social return in terms of accessibility and/or affordability.

*The objective of Project ARI is to implement a CAR-T development platform and make it available to patients in Spanish Hospitals that need them at strict production cost. (Contractual language as read by interviewee.)*

Affordability seemed to be a priority for interviewees, at least in the national context. Nevertheless, it is unclear if the funding and license agreements with institutions outside Spain included clauses on affordability of the treatments.

#### Transparency

During the interviews we asked for data on total investments received, costs to develop ARI-0001, regulatory costs, pre-clinical and clinical trial costs, as well as access to the funding and license agreements, but interviewees were reluctant to share this data. The high costs of regulatory processes were mentioned in several interviews as a problem, and one of the main drivers of high prices of CAR-T therapies. However, without concrete data, it was impossible to analyze the validity of these claims.

Regarding transparency of their research activities, HCB acted as a research center, publishing the results of their research in peer reviewed journals.

### Rules and norms in academic development of CAR-T therapies

The HE clause was seen by Spanish regulators, implementers, and civil society organizations interviewed as a steppingstone in the process of obtaining centralized approval at the EU level, and as a mechanism to incentivize academic innovation. The ‘Spanish model’ [26] was based on the establishment of more stringent regulatory requirements than in other EU MS [28, 35], including inspections and certification of good manufacturing practices (GMP), generation and reporting of safety and efficacy data through preclinical and preliminary data from clinical studies, the establishment of pharmacovigilance plans, and annual reporting of data. This interpretation of the HE aimed to ensure safety, efficacy, and quality of academic ATMPs while creating the conditions that orient these academic institutions towards centralized approval.

These stringent requirements and the need to speak the “regulatory language” when reporting to the regulator were highlighted as a challenge by some of the implementers interviewed within and outside HCB (CS_1, CS_2, CS_4, CS_7, CS_12), considering them as unnecessary and expensive.

#### Social norms support making CAR-T developed in “La Pública” accessible

Almost all Spanish interviewees highlighted ARI-0001’s development in the public healthcare system as a matter of pride, as well as its importance to understand its relationship with the treatment’s accessibility and affordability (CS_2, CS_3, CS_5, CS_7, CS_8, CS_9, CS_10, CS_11, CS_20).

The Spanish healthcare system is rooted in principles of universality, accessibility, and equity [63, 64]. Mostly delivered through public healthcare facilities (“La Pública”), with broad coverage and mostly free at the point of care, the healthcare system is valued very positively among its users [65]. The origins of the current healthcare system during the democratic transition and entrance to the EU (1986) might have helped to characterize it as a key feature of the modern welfare system [52]. This created shared social norms around the role of privatization and profiting from health-related goods and services.

These social norms emerged frequently throughout the interviews, with interviewees linking ARI-0001’s development in a hospital of the National Healthcare System with its accessibility and affordability:

*The fact of working in a public institution*,* of being doctors of ‘la Pública’ and having dedication to ‘la Pública’ and wanting to reach the maximum number of patients with a more reasonable price. Obviously*,* this had absolute relevance in how we have developed the ARI project. - CS_10*

These social norms helped align other actors beyond HCB with the mission of providing accessible and affordable pharmaceutical products, as the shared norms are reflected in other interviewees such as philanthropic funders or regulators. For one interviewee, the fact that these “living drugs” are made with patients’ cells, in public hospitals, justifies the need to have access to these therapies.

*We also understand that if we [the public healthcare system] participate [in the development]*,* and patients participate as well […]*,* if a therapy is developed from the patient’s cells*,* it is obvious that patients should receive those benefits.- CS_20*

#### “Public R&D? this is not Cuba!”

The perceptions from policy makers and other actors around the development of ARI-0001 in the public sector shifted considerably since the start of the project, from an initial skepticism about the hospital’s R&D capacity, to a more collaborative and supportive position as the development of ARI-0001 progressed.

*A high-profile person in the Health Department [regional Health office] asked me if we wanted to be like Cuba and produce our own medicines. […] luckily*,* the Department understood later that they needed to change that attitude and the subdirector of the department supported us*,* which was essential for the [change of attitude] - CS_10*

Interviewees also reported good relations and understanding with members of the Ministry of Health and the regulatory agency since the beginning (CS_1, CS_2, CS_7, CS_10, CS_12). However, some HCB researchers (CS_3, CS_10) perceived that they had to fight opposing views and criticism, being seen as “intruders” or “playing being Pharma” by entering a field that was not usually the realm of academic or healthcare institutions.

*’What are you doing?’ ’What you do is not innovative at all*,*’ ’it’s a second-generation CAR-T like so many others…’*,* ’why do you play being Pharma when you are not’. […] [It comes] from all sides! All the time since we started. Especially from the companies*,* but everyone. So*,* of course*,* the only way things are going to change is if they see that we can do it just like Pharma. - CS_3*

## Discussion

**Fig. 3:**
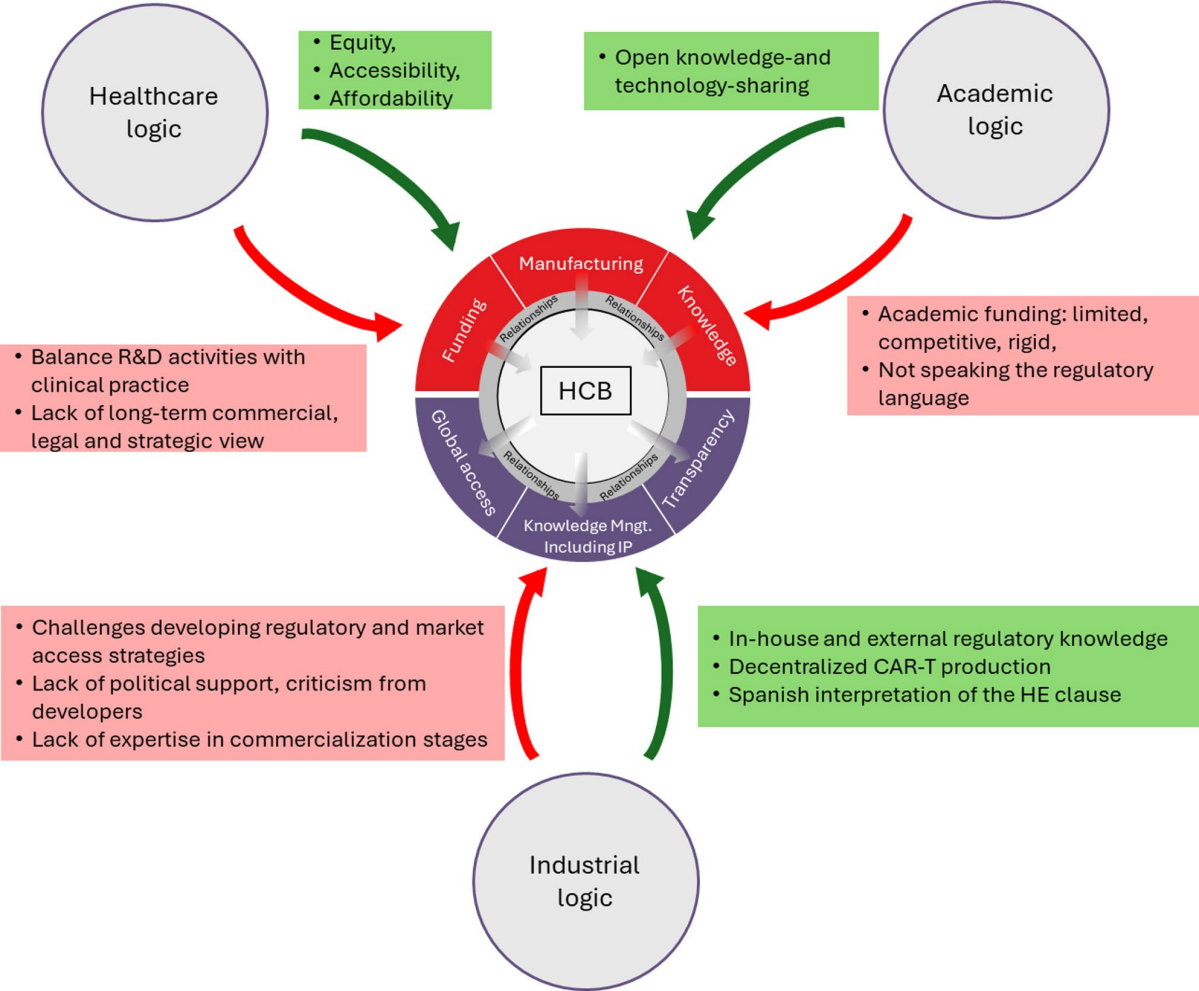
Challenges (in red) and strengths (in green) identified in HCB’s innovation model, derived from healthcare, academic and industrial logics. Note: the figure does not represent an exhaustive definition of each logic, but rather the specific characteristics from each logic that were mentioned during the interviews

We argue that the development of ARI-0001 took place as part of three distinct, often competing, logics [47] (Fig. 3). As a hospital which is part of the healthcare system, HCB operated under a healthcare logic. As an academic research center, it functioned under an academic logic. As ARI-0001’s development progresses HCB is also entering an industrial logic. The strengths, weaknesses, and innovative aspects of HCB’s innovation model stem from its capacity to span the boundaries of these logics [47].

Tellingly, interviewees from HCB did not consider many of their practices as innovative. Sharing and acquiring knowledge through open collaboration was seen as something relatively common in the medical academic field (academic logic). Providing affordable access was seen as common sense by many interviewees in the context of a public hospital (healthcare logic). Acquiring and speaking the “regulatory language” was deemed necessary and essential, but not an innovative feature of HCB’s model for the two members of the hospital team with regulatory expertise (industrial logic). These resources and practices in isolation, considered under separate logics, were not perceived as innovative; but put together they become an alternative innovation model.

HCB’s mission and practices (e.g., not seeking profits, expanding access to affordable care, and addressing a medical need) are deeply embedded in the values of the Spanish healthcare system. These values are perceived as a broader societal norm in Spain but become innovative when seen within the industrial logic of mainstream pharmaceutical R&D, which seeks to maximize profits and where objectives and practices like HCB’s are unusual.

This also posed challenges. Balancing clinical practice with the development of ARI-0001 was difficult, and the lack of a long-term regulatory or commercial strategy to set research priorities or learning how to deal with external sets of rules (e.g., regulatory standards, intellectual property) was challenging for HCB.

As a research hospital, openly transferring and acquiring knowledge was perceived as natural for HCB, as it facilitates research, reduces inefficiencies, and is seen as a way of gaining academic reputation [47]. Openly sharing knowledge and technology with partners and expanding access to ARI-0001 was therefore considered normal within this logic. It only becomes alternative, when seen from the mainstream pharmaceutical R&D system, where knowledge and technology is seen as a competitive advantage, and is therefore protected from competitors through secrecy, IP and other exclusivity rights.

However, the rigidity, competitiveness, and limited availability of academic funding posed barriers to access financial resources in time and quantity, although this was addressed by the flexibility and catalytic effect of philanthropic funding and the crowdfunding campaign. Another big challenge was adapting and complying with regulatory standards and practices as well as “speaking the regulatory language,” which differs from the “academic language.”

HCB faced challenges mobilizing and obtaining resources to fund late-stage development, or develop regulatory and market access strategies, all of which traditionally belong to the industrial logic, and have usually been carried out by larger pharmaceutical companies [9, 12, 17, 18] in profitable markets such as oncology [5, 6, 66]. This also explains the lack of support from some policymakers, and criticism by companies, who viewed HCB through a mainstream industrial logic and perceived pharmaceutical companies as the only actors that could develop and commercialize pharmaceutical products [67].

HCB tried to overcome these challenges by using its in-house regulatory knowledge, and through its relationship with the regulatory agency, which facilitated the adoption of elements of the industrial logic. Furthermore, the production of CAR-T therapies favors decentralized networks [20], which allows academic hospitals to leverage academic or hospital networks.

We argue that the Spanish interpretation of the hospital exemption clause created bridges between these three logics [47], building on their strengths while mitigating their challenges. By requiring industrial quality and reporting standards from academic developers, the HE facilitated the development of pharmaceutical innovations in healthcare systems geared towards providing equitable, accessible and universal care [26]. Working in academic and healthcare networks facilitated the sharing of knowledge and technology at national and international level, making the incorporation of quality and reporting standards widely accepted, enabling collaborations to facilitate global access, and reducing the costs of centrally manufactured CAR-T therapies. Operating as a part of the healthcare system allowed HCB to access its resources more easily (e.g., patients for clinical trials), which also reduces costs.

HCB’s alternative innovation model emerges in a market (rare disease and oncology) which has proven to be potentially highly profitable. This is distinct from other areas where alternative innovation models have emerged due to the absence of a sufficiently profitable market, such as in antimicrobial resistance (AMR) or neglected diseases of poverty [6, 68]. It suggests that even in lucrative areas, alternative innovation models can offer advantages over the mainstream market-oriented model.

## Conclusions

The development of ARI-0001 shows the potential of research hospitals to develop more affordable therapies for rare diseases and increase knowledge- and technology-sharing to expand local production and broaden access.

The hospital exemption clause is a successful regulatory tool that can enable the EU to harness all the potential of its academic, healthcare and industrial infrastructure, boosting more affordable pharmaceutical innovation in the region. Additionally, the HE is a successful regulatory tool that could be potentially adopted by other countries and regions.

Our research also highlights some of the key challenges to fully develop this alternative innovation model. Adjusting and creating new funding schemes to cover the late clinical stages of development and regulatory costs is essential to support academic developers. Regulators could also waive or reduce fees, provide regulatory advice, and facilitate the acquisition of regulatory expertise for academic developers. The creation of new, and strengthening of existing governance mechanisms, to coordinate an academic network of ATMP developers could facilitate the expansion of this model.

Finally, although societal norms regarding affordability and access to healthcare in Spain shaped HCB’s practices, equitable global access is not embedded by design in this model. With ongoing discussions at national and EU levels to expand public involvement in all segments of the pharmaceutical value chain [69], policymakers and funders can learn from different experiences in other fields of pharmaceutical R&D to include a global health perspective, ensuring that access to the fruits of scientific progress are globally shared in an equitable way.

Some organizations active in other therapeutic areas include global access provisions, plans and/or principles in their collaborations and funding agreements to ensure that their investments provide guidance on how to establish affordable pricing policies, increased transparency, and/or facilitate global knowledge and technology sharing [70–73].

EU governing actors (e.g., governments, EU Commission) and funders should aim to expand international collaborative academic networks to conduct clinical trials and manufacture products. The experience of different Product Development Partnerships as coordinators or “orchestra conductors” of international networks of R&D actors [74, 75], initiatives like the Global Training Hub for Biomanufacturing created by the World Health Organization and the International Vaccine Institute [76] or the mRNA vaccine technology transfer program [77], that aim to expand production capacities and access to these breakthrough therapies in LMICs, could be valuable examples to learn from and bring into the rare disease niche.

Our study has several limitations that point to areas for further study. Firstly, interviews did not cover HCB’s market access strategy, which is a limitation of academic CAR-T developers. Secondly, we did not focus our analysis on the role of intellectual property (IP) rights, as they were not a point of emphasis in the interviews. IP has been described as a key barrier to access in the literature, so future research could delve more deeply into how IP was managed in this case and how it may influence academic development of advanced therapies. Thirdly, lack of access to data on costs of development, production and price setting is a major limitation of our study, impeding comparisons between ARI-0001 and other commercial CAR-T therapies available in the market. Future research should address the costs of developing these drugs, as well as how these costs influence final prices. Finally, while this model reflects the specific characteristics of CAR-T therapies (e.g., individualized and networked production, and the existence of the hospital exemption clause), and some of its features cannot be extrapolated, some of its lessons extend beyond this particular case. The use of regulatory flexibilities to enable an alternative innovation pathway, the capacity of academic institutions to operate downstream in development if supported by the catalytic role of public and philanthropic financing, illustrate mechanisms that may be transferable to other advanced therapy medicinal products and certain areas of rare-disease research. ARI-0001’s development shows the capacity of alternative innovation models to deliver innovation and access to more affordable treatments in a cutting-edge technology and a highly lucrative market. We expect that our insights can help clarify the challenges and opportunities that this model presents and help shed light on how to harness its full potential.

## Supplementary Information

Below is the link to the electronic supplementary material.

Supplementary Material 1

## Data Availability

Where consent was obtained, the interview transcripts used and/or analyzed during the current study will be made available on an open data repository. A temporary link can be found here: 10.5281/zenodo.13384129.

## Abbreviations

AEMPS: In Spanish: Spanish Agency of Medicines and Medical Devices
ATMP: Advanced Therapy Medicinal Products
CAPIS: Complex Adaptive Pharmaceutical Innovation System
CAR-T: Chimeric antigen receptor T-cell
CDTI: In Spanish Centre for the Development of Industrial Technology
CERTERA: In Spanish National Network Consortium for the Development of Advanced Therapies
EMA: European Medicines Agency
GMP: Good Manufacturing Practices
HCB: Hospital Clínic de Barcelona
HE: Hospital exemption
HOVON: In Dutch Foundation Hemato-Oncology for Adults Netherlands
IDIBAPS: In Catalan Clínic Barcelona Research Foundation - Institute of Biomedical Research August Pi i Sunyer
IP: Intellectual Property
MS: Member State
NBM: New business models for governing innovation and global access to medicines
OS: Overall Survival
PERTE: In Spanish Strategic Project for Recovery and Economic Transformation
PFS: Progression-Free Survival
PRIME: European Medicines Agency Priority Medicines program
R&D: Research and Development
R/R ALL: Relapsed or refractory acute lymphoblastic leukemia
TERAV: In Spanish Red Española de Terapias Avanzadas
